# Terrestrial Birth and Body Size Tune UCP1 Functionality in Seals

**DOI:** 10.1093/molbev/msae075

**Published:** 2024-04-12

**Authors:** Michael J Gaudry, Jane Khudyakov, Laura Pirard, Cathy Debier, Daniel Crocker, Paul G Crichton, Martin Jastroch

**Affiliations:** Department of Molecular Biosciences, The Wenner-Gren Institute, Stockholm University, Stockholm, Sweden; Department of Biological Sciences, University of the Pacific, Stockton, CA, USA; Louvain Institute of Biomolecular Science and Technology, Université catholique de Louvain, Louvain-la-Neuve, Belgium; Louvain Institute of Biomolecular Science and Technology, Université catholique de Louvain, Louvain-la-Neuve, Belgium; Department of Biology, Sonoma State University, Rohnert Park, CA, USA; Biomedical Research Centre, Norwich Medical School, University of East Anglia, Norwich, UK; Department of Molecular Biosciences, The Wenner-Gren Institute, Stockholm University, Stockholm, Sweden

**Keywords:** UCP1, brown adipose tissue, nonshivering thermogenesis, pseudogene, pinniped

## Abstract

The molecular evolution of the mammalian heater protein UCP1 is a powerful biomarker to understand thermoregulatory strategies during species radiation into extreme climates, such as aquatic life with high thermal conductivity. While fully aquatic mammals lost UCP1, most semiaquatic seals display intact *UCP1* genes, apart from large elephant seals. Here, we show that UCP1 thermogenic activity of the small-bodied harbor seal is equally potent compared to terrestrial orthologs, emphasizing its importance for neonatal survival on land. In contrast, elephant seal UCP1 does not display thermogenic activity, not even when translating a repaired or a recently highlighted truncated version. Thus, the thermogenic benefits for neonatal survival during terrestrial birth in semiaquatic pinnipeds maintained evolutionary selection pressure on UCP1 function and were only outweighed by extreme body sizes among elephant seals, fully eliminating UCP1-dependent thermogenesis.

## Introduction

Uncoupling protein 1 (UCP1) catalyzes mitochondrial proton leak in mammalian brown adipose tissue (BAT), thereby accelerating uncoupled respiration for nonshivering thermogenesis (NST; Oelkrug et al. 2015). As this heat-producing mechanism likely facilitated species expansion into thermally challenging niches, UCP1 provides a potent biomarker to trace the evolution of mammalian thermoregulation. In terrestrial mammals and in humans, UCP1 has been intensively investigated for biomedicine as a potential therapeutic target for cardio-metabolic diseases, but questions remain regarding physiological relevance, molecular mechanisms (Crichton et al. 2017), and the selective forces governing UCP1 evolution. Indeed, little is known about how UCP1 may have adapted to suit differing thermoregulatory demands dictated by various ecophysiological constraints.

**Fig. 1.**
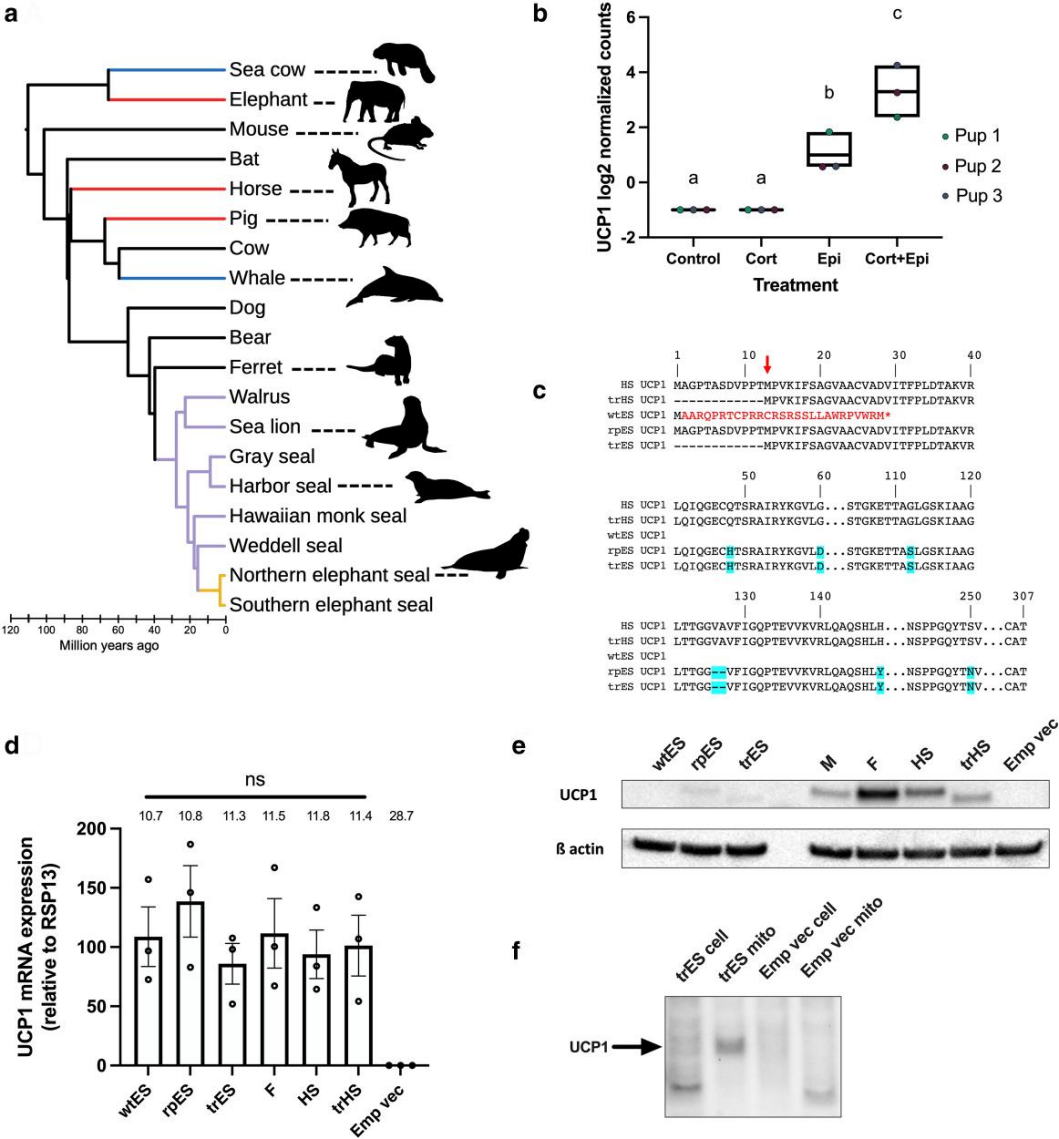
*UCP1* transcripts from ex vivo northern elephant seal blubber suggest possible UCP1 variants. a) Phylogeny (modified from Berta et al. 2018 and Yuan et al. 2021) highlighting the loss of UCP1 among fully aquatic mammals (sea cow, whale in blue) and large-bodied terrestrial mammals (elephant, horse, pig in red). The pinniped lineage (walrus, sea lion, gray seal, harbor seal, hawaiian monk seal, weddell seal) is highlighted in purple with northern and southern elephant seals in yellow. Animal silhouettes are public domain from phylopic.org under Creative Commons licenses. b) Expression of *UCP1* mRNA in blubber tissue slices from weaned northern elephant seal pups (*n* = 3) treated with cortisol (Cort), epinephrine (Epi), or both. Different letters denote significant differences (*P* < 0.05). Box boundaries denote maximum and minimum, and mean (internal line). c) UCP1 alignment highlighting the elephant seal UCP1 frameshift mutation (wtES; red), repaired (rpES), and potentially rescuing truncation (trES; red arrow) put forward by Yuan et al. (2022) with highlighted AA residue substitutions relative to harbor seal (HS) and truncated harbor seal (trHS) UCP1 (cyan). d) *UCP1* mRNA expression of transiently transfected HEK293 cells (*n* = 3) with seal and ferret (F) variants, as well an empty vector control (Emp vec). Error bars denote SEM, and *UCP1* Ct values are above each bar. e) Representative western blot of HEK293 cells transfected with seal, ferret, and mouse (M) UCP1 variants, as well an empty vector control. f) Western blot of whole cell lysate (cell) versus isolated mitochondrial fraction (mito) from HEK293 cells transfected with trES UCP1 and an empty vector control.

Despite heavy reliance on UCP1-mediated NST for many eutherian mammals, the *UCP1* gene has been repeatedly inactivated throughout the eutherian radiation, in fully aquatic lineages (whales and sea cows), and predominantly in large-bodied terrestrial species (e.g. elephants, horses, and pigs; Fig. 1a; Gaudry et al. 2017). High thermal conductance of water seemingly favors heat retention mechanisms (e.g. blubber and countercurrent heat exchangers) rather than acute heat production strategies such as UCP1-dependent thermogenesis (Gaudry et al. 2017; Yuan et al. 2021). Surprisingly, however, many semiaquatic pinnipeds have maintained an intact *UCP1* gene (Gaudry et al. 2022). *UCP1* gene expression and protein detection in newborn harbor seals (*Phoca vitulina*; Sakurai et al. 2015) suggest importance for terrestrial birth, but thermogenic functionality of seal UCP1 has never been verified. Further adding confusion to the existence of classical NST in semiaquatic mammals, a novel potential *UCP1* pseudogene was identified in the common ancestor of northern and southern elephant seals (*Mirounga angustirostris* and *Mirounga leonina*; Fig. 1a; Gaudry et al. 2022). Elephant seals are the largest pinnipeds, with adult males reaching over 2 t (Deutsch et al. 1994). Large body size reduces surface area to body volume ratios, thereby retaining heat and possibly reducing the need for thermogenesis. This notion is supported by the inverse relationship between body size and NST capacity (Oelkrug et al. 2015).

Elephant seals display a single nucleotide frameshift deletion immediately following the start codon (Gaudry et al. 2022). Yuan et al. (2022) contested the pseudogenization of elephant seal *UCP1*, speculating that an alternative start site 12 codon triplets downstream of the recognized *UCP1* start codon would produce a truncated, but functional protein. Pending verification of thermogenic functionality of seal UCP1 and its inactivation in large elephant seals, the outcome has major implications for determining the hierarchy of evolutionary forces acting on UCP1-dependent thermogenesis: the differences in selection pressures dictated by aquatic versus semiaquatic lifestyles and the potentially overarching effects of body size on thermoregulation. We utilized experimental systems and new structural insights of UCP1 (Jones et al. 2023) to delineate the existence of UCP1-dependent thermogenesis in seals.

## Results

We first confirmed *UCP1* transcription in northern elephant seal pup blubber slices upon ex vivo epinephrine and cortisol stimulation (Fig. 1b). Transcripts retained the 1 bp frameshift deletion expected from the genomic data (Gaudry et al. 2022). This suggested three possibilities of translating elephant seal UCP1: (i) a frameshifted 28 amino acid (AA) truncated peptide, (ii) a frameshift read-through (via described mechanisms; Wang et al. 2022) producing an in-frame 305 AA protein, or (iii) use of the proposed alternative start site (Yuan et al. 2022) creating a 293 AA protein with an N-terminal truncation (Fig. 1c).

We then transiently overexpressed UCP1 variants for functional respirometric analyses in HEK293 cells, which produced similar *UCP1* mRNA levels across variants (Fig. 1d), allowing equal opportunity for each variant to be overexpressed. Mouse (*Mus musculus*), ferret (*Mustela putorius furo*), and harbor seal UCP1 are clearly detected with variable signals, given potential differences in epitopes and antibody cross-reactivity (Fig. 1e). For wildtype elephant seal UCP1, which retained the 1 bp frameshift deletion immediately following the start codon, neither full-length nor truncated protein versions could be detected, indicating that N-terminal truncation does not typically occur under unforced conditions.

**Fig. 2.**
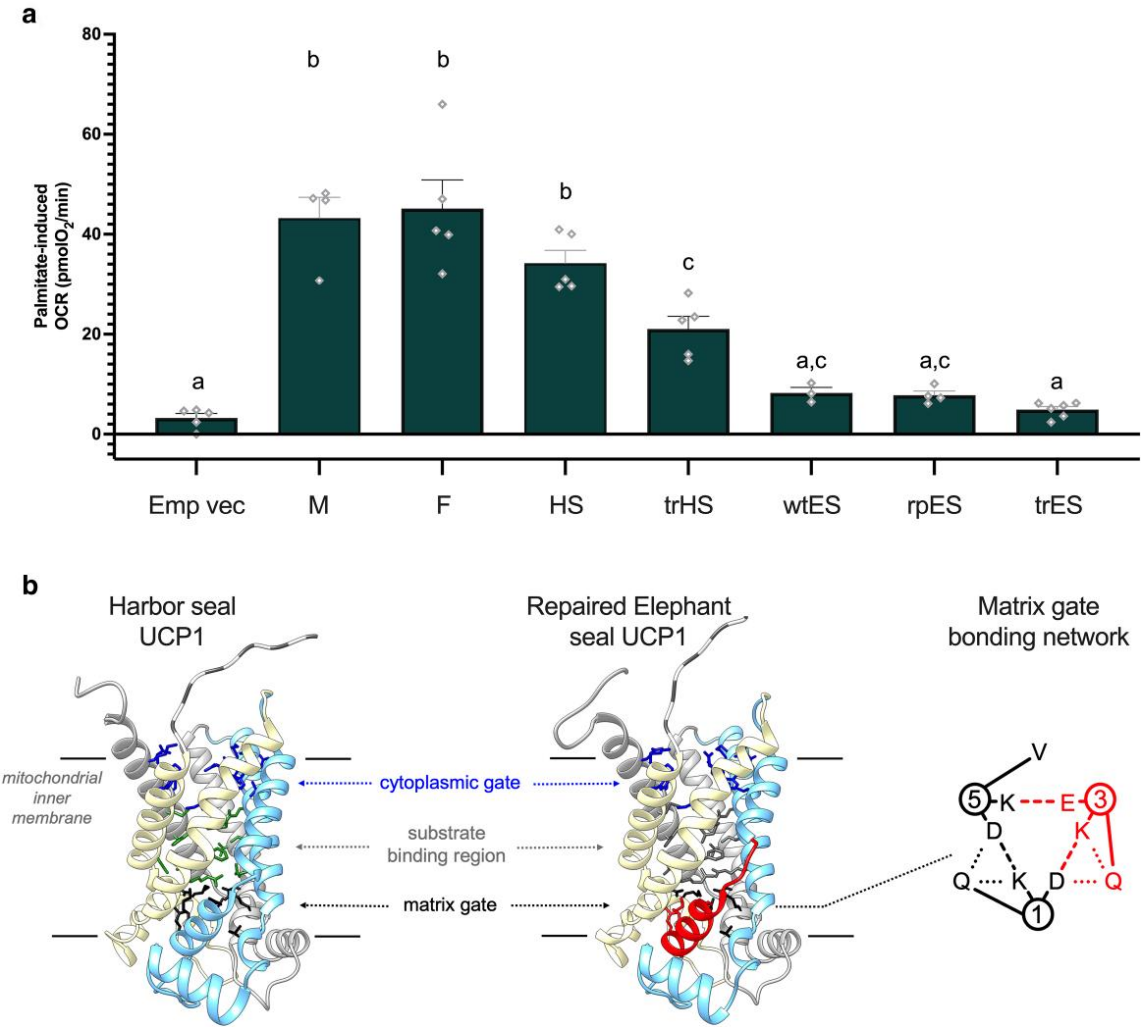
a) Cross-species comparison of palmitate-induced (100 µM) OCRs of transfected HEK293 cells confirming that elephant seal UCP1 is not thermogenic. OCRs have been corrected for nonmitochondrial respiration. Data are mean ± SEM. *N* = 3 to 6 from 3 independent runs. Statistical significances are denoted by independent letters in *P* < 0.05, one-way ANOVA with Tukey's post hoc test (see supplementary fig. S1, Supplementary Material online, for full respirometric traces). UCP1 variants are abbreviated as mouse (M), ferret (F), harbor seal (HS), truncated harbor seal (trHS), wildtype elephant seal (wtES), repaired elephant seal (rpES), and truncated elephant seal (trES), along with the empty vector control (Emp vec). b) Harbor seal versus repaired elephant seal UCP1 homology structure analyses. N-Terminal truncated AAs are indicated with dashed residues. Compromised elephant seal helix 3 and associated substrate binding region and matrix gate interactions are highlighted in red caused by V126-A127 deletion.

UCP1 of the harbor seal (a small-bodied seal), mouse, and ferret (a phylogenetic outgroup of pinnipeds; Fig. 1a) all displayed similar oxygen consumption rate (OCR) increases upon injection of the canonical UCP1 activator (palmitate; Fig. 2a; supplementary fig. S1, Supplementary Material online), demonstrating for the first time that small semiaquatic pinnipeds have retained a thermogenic UCP1, matching activity levels of small terrestrial eutherians. These data are in line with potential BAT and UCP1 expression in newborn harbor seals that diminishes with age (Sakurai et al. 2015), underscoring the importance of this thermoregulatory mechanism for the defense of neonatal body temperatures soon after birth while on land.

Wildtype elephant seal UCP1-transfected HEK293 cells failed to increase OCRs in response to palmitate above empty vector levels (Fig. 2a; supplementary fig. S1, Supplementary Material online). Next, to simulate a frameshift mutation read-through, we repaired the 1 bp frameshift mutation in elephant seal UCP1. This mutation repair failed to elicit a UCP1-mediated thermogenic response to palmitate (Fig. 2a; supplementary fig. S1, Supplementary Material online).

We sought to verify that N-terminal truncation per se is a plausible strategy in seals to circumvent inactivating mutations in the 5′ region of exon 1. Therefore, we truncated both harbor seal and elephant seal UCP1 using the alternative start site suggested by Yuan et al. (2022). Truncation of seal UCP1 reduced protein levels compared to the full-length variant (Fig. 1e), possibly affected by inevitably varying the Kozak sequence for ribosomal recognition from the typical guanine (G) at position +4 to a cytosine (C) in the truncated versions (Kozak 1997). Nevertheless, truncated harbor seal UCP1 retained robust dose-dependent OCR responses to palmitate (Fig. 2a; supplementary fig. S1, Supplementary Material online), demonstrating that the use of the alternate start site can produce a functionally competent protein. By contrast, truncated elephant seal UCP1 provided detectable protein that still localized to the mitochondria (Fig. 1f), but fully lacked thermogenic responses to palmitate (Fig. 2a). This indicated that only seven AA substitutions dramatically erase elephant seal protein activity (Fig. 1c). Using the recently determined human UCP1 structure (Jones et al. 2023), we performed structural homology modeling. The harbor seal UCP1 retains all key features, but the unique V126-A127 deletion in elephant seal UCP1 is projected to compromise transmembrane helix 3 formation and associated substrate binding and matrix gate interactions (Fig. 2b), negatively impacting protein folding, stability, and function.

**Fig. 3.**
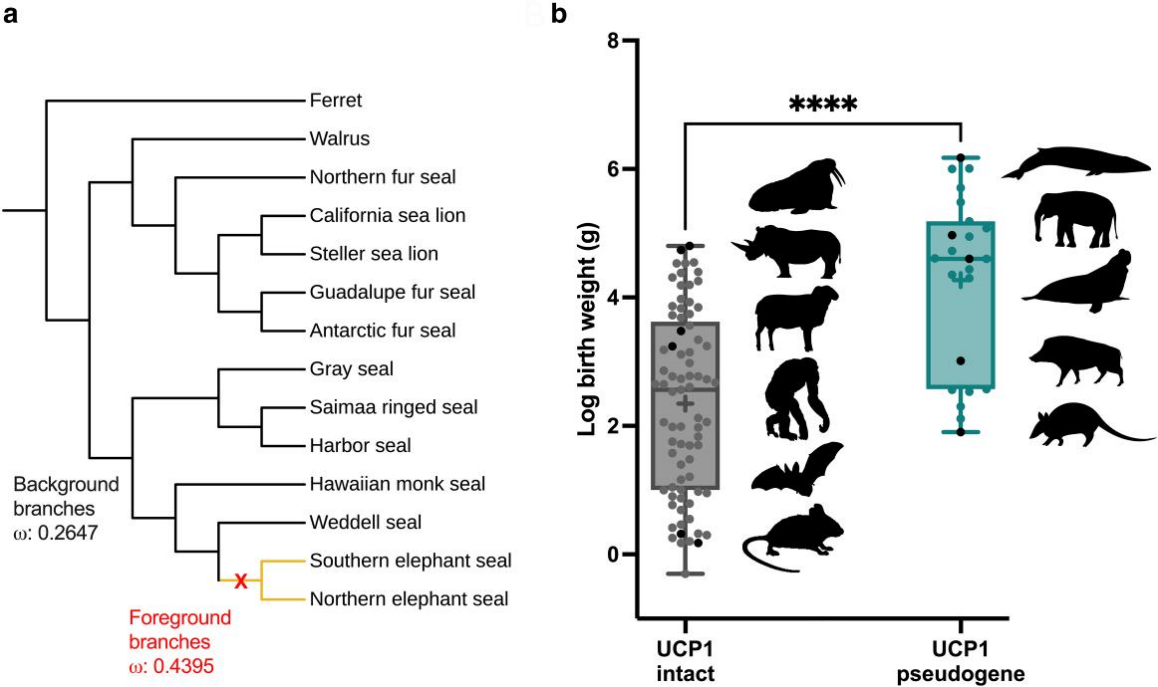
a) Selection pressure analysis of *UCP1* coding sequences among pinniped species. Global selection pressures have been calculated for background (black; non-elephant seal) branches with intact *UCP1* genes and foreground (yellow; elephant seal) branches displaying inactivating mutations. b) Log-transformed birth weights of eutherian species with intact *UCP1* genes versus those that display *UCP1* pseudogenes. Black dots correspond to the neighboring animal silhouette in order of size. Animal silhouettes are public domain from phylopic.org under Creative Commons licenses. Box and whisker plots denote the median values (line), 25th to 75th percentiles, and mean (+). The whiskers extend to the maximum and minimum values. An unpaired *t*-test was performed, and **** denotes *P* < 0.0001.

We then performed selection pressure analyses on *UCP1* coding sequences from available pinnipeds, revealing that the elephant seal *UCP1* pseudogenes appear to have evolved at a higher nonsynonymous to synonymous substitution rate (dN/dS or *ω* = 0.44) compared to all combined background branches (*ω* = 0.26; Fig. 3a). To further examine the linkage between body size and functional UCP1 loss, we collected birth weights from eutherian species with previously described intact or inactivated *UCP1* genes (Gaudry et al. 2017). On average, eutherian species with *UCP1* pseudogenes have higher birth weights than those that retain intact *UCP1* loci (Fig. 3b).

## Discussion

Widespread genetic conservation of UCP1 in semiaquatic seals (Gaudry et al. 2022) and functional confirmation in harbor seals highlights the importance of UCP1-mediated NST for neonatal survival on land compared to fully aquatic eutherian lineages (cetaceans and sirenians) that have lost UCP1. In elephant seals, in which body size has peaked in the pinniped lineage, our experiments reveal that UCP1 thermogenic function has been lost, strongly supporting the overarching concept that increased body size is a main driver to eliminate UCP1-mediated NST (Gaudry et al. 2017). Northern elephant seal neonates are ∼38 kg at birth and remarkably gain ∼4 kg/day until they are weaned at around 25 days at ∼132 kg (Deutsch et al. 1994), which is perhaps sufficient to maintain homeothermic body temperatures without UCP1. By contrast, harbor seal pups are relatively small at birth (∼11 kg) and gain nearly 0.4 kg/day until they are weaned at ∼32 days and a weight of ∼24 kg (Cottrell et al. 2002), retaining the need for UCP1-mediated NST. On average, eutherian mammals with *UCP1* inactivations display higher weights at birth (Fig. 3b), the period when BAT-mediated thermogenesis is most crucial to defend body temperatures against cold extrauterine environments, compared to those that maintain intact *UCP1* genes. Differences in muscle mass, muscle NST, or other UCP1-independent mechanisms likely also contribute to variable UCP1 selection pressures (Roesler and Kazak 2020; Wright et al. 2021).

Notably, some species lack functional UCP1 despite their small size (e.g. sloths, armadillos, and pangolins), which may be related to their low metabolic rates and/or calorically poor diets and may explain their modern confinement in tropical ecological niches (Gaudry et al. 2017). On the other hand, some large-bodied eutherians (e.g. rhinoceroses, bovids, and even walruses) display intact *UCP1* genes though the functionality of these protein variants has in most cases not been examined. Similarly, little, if anything, is known regarding the expression of BAT and its thermogenic contribution to the defense of body temperature in many of these large-bodied mammals. It should be noted that birth weights of modern eutherians do not necessarily paint a complete picture since these UCP1 inactivations occurred millions of years ago, and extensive estimates would have to be conducted for the extinct ancestors of these lineages.

Selection pressure analyses were highly consistent with those performed by Yuan et al. (2022). While elephant seal branches display higher *ω* values than relatives with intact *UCP1* genes (Fig. 3a), *ω* = 0.44 is lower than would be expected (*ω* = 1) for an inactivated gene undergoing neutral evolution. Similar patterns of selection pressure have been noted for equid *UCP1* pseudogenes for instance (Gaudry et al. 2017) and could be indicative of a relatively recent pseudogenization event in the *Mirounga* (elephant seal) lineage with inadequate time to accumulate sufficient random mutations for *ω* = 1. Indeed, phylogenetic bracketing suggests this inactivation likely occurred in a common ancestor of modern elephant seals between 2.5 and 15 million years ago (Berta et al. 2018; Gaudry et al. 2022).

Ecophysiological factors are likely to be inherently linked to the loss or retention of UCP1-mediated NST. Harbor seal pups lose their lanugo (insulating natal pelage) prior to birth and spend >50% of their time nursing while in the water (Oftedal et al. 1991; Schreer et al. 2010; Favilla and Costa 2020). While this presents a significant thermoregulatory challenge, the pups also display BAT (Sakurai et al. 2015). In contrast, elephant seal pups retain their lanugo throughout postnatal development and generally do not enter water until after weaning, when adiposity reaches >40% (Rea and Costa 1992; Noren 2002; Hammill 2018). Northern elephant seals seek mild climates to give birth and molt, migrating vast distances from foraging grounds, as far north as the Aleutian Islands, Alaska, to return to beaches ranging from central California, United States, to central Baja California, Mexico, during the winter months (Stewart et al. 1994; Le Boeuf et al. 2000). Historically, southern elephant seals also utilized mild temperate habitats (South Africa, Tasmania) for breeding and molting, although current rookeries are mainly restricted to subantarctic islands (de Bruyn et al. 2009; Helm et al. 2022). In comparison, harbor seals do not migrate prior to breeding and molting and have a broad distribution that includes the northern latitudes of North America, Asia, and Europe; the northernmost harbor seal subpopulations are found in Greenland, Iceland, and Svalbard (Andersen et al. 2011; Blanchet et al. 2021).

Taken together, the evolutionary fate of pinniped UCP1 variants underscores the hierarchy of driving forces acting on UCP1-dependent thermogenesis in mammals: (i) Fully aquatic life with constantly high thermal conductance of water favors strategies that increase heat retention and relaxes constraints on UCP1, as found for sirenians and cetaceans; (ii) semiaquatic life with early development on land requires typical neonatal UCP1-dependent thermogenesis for most pinnipeds; and (iii) large body size reduces heat loss due to decreased surface area to volume ratios and likely increases muscle thermogenesis, which thus relaxes constraints on UCP1 retention as found for elephant seals.

## Materials and Methods

### Sample Collection

Northern elephant seals (*M. angustirostris*) were sampled at Año Nuevo State Reserve (San Mateo County, CA, United States). All animal handling procedures were approved by the Sonoma State University Institutional Animal Care and Use Committee and were conducted under National Marine Fisheries Service Marine Mammal Permits 14636, 19108, and 23188. Blubber samples were collected from anesthetized weaned pups and juveniles as described previously (Deyarmin et al. 2019; Debier et al. 2020).

### Ex Vivo Blubber Slices and Epinephrine Stimulation

Blubber tissue was precision cut into 1-mm-thick slices and cultured with shaking in culture media for 48 h as recently described (Kashiwabara et al. 2023). Blubber slices were treated with either epinephrine (100 nM during the last 12 h of culture), cortisol (2 μM cortisol throughout 48 h of culture), and both cortisol and epinephrine (2 μM cortisol for 48 h and 100 nM epinephrine for the last 12 h of culture); no hormones were added to control slices. After 48 h, all blubber slices were rinsed in PBS, flash-frozen, and stored at −80 °C for RNA extraction.

### RNA Sequencing

RNA isolation from blubber samples, library preparation, and RNA sequencing were conducted as previously described (Deyarmin et al. 2019; Khudyakov et al. 2022; Kashiwabara et al. 2023). Briefly, transcriptomes were assembled de novo using Trinity (Haas et al. 2013) and annotated using DIAMOND (Buchfink et al. 2021). Transcript abundance quantification and differential expression analyses were conducted using Salmon (Parto et al. 2017) and DESeq2 (Love et al. 2014), respectively. *UCP1* transcript counts were normalized by estimated library size, and a pseudocount of 0.5 was added to facilitate plotting.

Trinity de novo RNA sequencing assemblies of *UCP1* from northern elephant seal blubber samples treated ex vivo with epinephrine and cortisol were aligned to northern elephant seal *UCP1* coding sequence inferred from the genomic data (PITE01002540.1) using Geneious 9 (supplementary data file S1, Supplementary Material online).

### Structural Modeling

Structural models of HS and elephant seal UCP1 were generated by AA sequence alignment and homology modeling to human UCP1 (>82% sequence identity; PDB structure: 8G8W; Jones et al. 2023) using UCSF Chimera (Pettersen et al. 2004).

### UCP1-Plasmid Preparation

Synthesized UCP1 coding sequence variants (Bio Basic Inc.) were cloned into pcDNA3.1 vectors at XhoI and XbaI cloning sites. Variants included ferret (*M. putorius furo*), HS (*P. vitulina*; full length and truncated), and northern elephant seal (*M. angustirostris*; wild-type, repaired, and truncated) UCP1 and were inferred from genomic data (accession numbers AEYP01069989.1; RXNX01005441.1; and PITE01002540.1). Empty and mouse UCP1-containing pcDNA 3.1 vectors were also used as negative and positive controls, respectively. Vectors were transformed in DH5a *E. coli*. (Invitrogen) according to the manufacturer's protocol and grown overnight on LB agar + 100 µg/mL ampicillin plates. Correct sequence identities were confirmed by Sanger sequencing. Clones were then picked and used to inoculate liquid LB cultures that were shaken at 225 rpm overnight at 37 °C. Plasmids were then purified using a Qiagen Miniprep Kit.

### HEK293 Cell Transient Transfection

HEK293 cells were grown in DMEM, high glucose (Gibco) supplemented with 10% fetal bovine serum (Gibco) and 1% penicillin/streptomycin (Gibco; stock: 10,000 U/mL) at 37 °C and 5% CO_2_. On the day of transfection, cells were trypsinized from T175 flasks using 0.05% trypsin-EDTA (Gibco) and counted in duplicate, and 1.76 × 10^6^ cells in 3.2 mL of growth medium were aliquoted into 15-mL falcon tubes. Transfection mixtures were prepared with 900 µL pure DMEM, high glucose, 9 µg plasmid DNA, and 36 µL PolyFect Transfection Reagent (Qiagen). After 15 min, transfection mixtures were applied to HEK293 cell aliquots. Cells were then seeded on Agilent Seahorse xf96 cell culture plates for respiratory experiments and on 12-well plates for protein and RNA isolation.

### Mitochondrial Isolation from HEK293 Cells

To verify mitochondrial localization, mitochondrial isolation was performed from HEK293 cells transfected with either trES UCP1 or an empty pcDNA3.1 vector. The cells were scraped from two 15-cm dishes, and mitochondria were isolated according to the protocol outlined in Jastroch (2012).

### Western Blot

Protein was isolated from transfected HEK293 cells using RIPA buffer (50 mM NaCl, 50 mM TRIS, 0.5% sodium deoxycholate, 1% IGEPAL CA-630, and 0.1% sodium dodecyl sulfate). Protein quantification was performed using Bradford reagent (Sigma-Aldrich). A total of 20 µg of protein was loaded into each well of Bolt 4% to 12% Bis-Tris Plus gels (Invitrogen) and electrophoresed according to the manufacturer's instructions using the Mini Gel Tank (Invitrogen). Transfers were performed using iBolt 2 NC Regular Stacks (Invitrogen) and the iBlot 2 Gel Transfer Device. Nitrocellulose membranes were reversibly stained with Ponceau and then blocked with 5% milk solution in TBS-T buffer for 1 h. Membranes were then incubated in 1:1,500 dilution of AB155117 rabbit polyclonal anti-UCP1 antibody (Abcam) in TBS-T with 5% bovine serum albumin (BSA, Sigma-Aldrich) overnight at 4 °C while shaking. Membranes were then washed with TBS-T, and an anti-rabbit secondary antibody was applied in 5% milk + TBS-T for 1 h. Blots were then imaged using Clarity Western ECL Substrate on a Bio-Rad ChemiDoc system. Blots were then stripped and blocked, and a 1:10,000 mixture β-actin HRP-linked antibody (Santa Cruz sc47778 Lot: F0215) and 5% milk + TBS-T was applied for 20 min before reimaging the blot.

### qPCR

RNA was isolated from transfected HEK293 cells using a RNeasy kit (Qiagen). RNA samples were quantified using a NanoDrop One Microvolume UV-Vis Spectrophotometer (Thermo Scientific), and cDNA synthesis was performed using a QuantiTect Reverse Transcription kit (Qiagen) according to the manufacturer's instructions. Custom UCP1 primers designed in Geneious to target conserved regions of ferret and seal UCP1 coding regions were used in qPCR to quantify UCP1 transcript levels. qPCR reactions were set up with SYBR Green JumpStart Taq ReadyMix (Sigma-Aldrich), 0.5 µM of each primer, and 5 ng of template cDNA. A cycling profile of 95 °C for 10 min, 95 °C for 15 s, and 60 °C for 1 min, for 40 cycles, followed by a melt curve where the samples are heated to 65 °C for 30 s and then the temperature ramped up at 0.5 °C/s for 60 cycles was conducted with a Bio-Rad CFX 384 Real-Time System C1000 Touch Thermal Cycler. Analysis was performed using CFX Maestro 4.1 software. The primer efficiencies were verified to be between 95% and 105%.

Primers used were as follows:

Seal_UCP1F3q 5′-TGGATGTGGTAAAAACCCGATT-3′.
Seal_UCP1R3q 5′-GCAAGAAGGAAGGTACAAATC-3′.
Human RSP13_F 5′-CTTGTGCAACACCATGTGAA-3′.
Human RSP13_R 5′-CCCCACTTGGTTGAAGTTGA-3′.

### Plate-Based Respirometry

After 24 h from the initial point of transfection, the cells seeded in the Agilent Seahorse xf96 cell culture plates were washed with XF assay medium supplemented with glucose (10 mM), pyruvate (10 mM), 0.4% essentially fatty acid-free BSA, and 2 mM glutamine. OCRs were measured on a Seahorse XFe96 Extracellular Flux Analyzer (Agilent) using 1 min mix, 2 min wait, and 3 min measure cycles. Baseline OCR was measured for three cycles, followed by injection of oligomycin (end concentration: 4 µg/mL) to inhibit ATP synthase where proton leak-linked respiration was measured for three cycles. The UCP1 activator palmitate (end concentration: 50 or 100 µM) or the vehicle control BSA solution was then injected, and OCR was measured for four cycles. The palmitate had been previously equilibrated with BSA at a 6:1 molar ratio (1 mM sodium palmitate:0.17 mM BSA). The injection of 2,4-dinitrophenol (DNP; end concentration: 100 µM), an artificial uncoupler, was then used to achieve the maximal OCR and measured for three cycles. Finally, OCRs were measured for three cycles following the injection of rotenone (end concentration: 4 µM) and antimycin A (end concentration: 2 µM), which were used to inhibit the electron transport chain. All OCR data were corrected for nonmitochondrial respiration. Palmitate-induced respiration was calculated by subtracting the average of all three measurement points following oligomycin injection from the first two time points following palmitate or vehicle control injection.

### Selection Pressure Analysis

UCP1 coding sequences were annotated and collected from accession numbers listed in supplementary table S1, Supplementary Material online. An alignment was performed using MUSCLE (Edgar 2004; see supplementary data file S2, Supplementary Material online). The phylogenetic tree was based on Berta et al. (2018) and Yuan et al. (2021). The codeml M2 model from the PAML 4.8 software package (Yang 2007) was used with *Mirounga* branches labeled as foreground branches.

### Birth Weight Analysis

Birth weights were collected from sources listed in supplementary table S2, Supplementary Material online, for eutherian species with described *UCP1* pseudogenes and described intact *UCP1* genes according to Gaudry et al. (2017). Birth weights were log transformed and graphed in box and whisker plots.

### Statistics

Changes in elephant seal *UCP1* expression in response to hormone treatment were assessed using linear mixed effects models with log_2_-transformed counts as a fixed effect and animal ID as a random effect using lme4 and lmerTest in R v4.1.0. Pairwise post hoc comparisons between treatments were conducted using the emmeans package (adjustment = Tukey).

Delta Ct values from qPCRs between variants as well as OCRs between UCP1 activator treatments were assessed to using an ordinary one-way ANOVA with a Tukey post hoc test in GraphPad Prism 9. The same program was used to perform an unpaired *t*-test between birth weights of eutherian species with either intact or inactivated *UCP1* genes.

## Supplementary Material

msae075_Supplementary_Data

## Data Availability

Data available on request.
