# Supplementary material for: Terrestrial Birth and Body Size Tune UCP1 Functionality in Seals
Source: Mol Biol Evol. 2024 Apr 12;41(4):msae075. doi: 10.1093/molbev/msae075 (PMC11050727; doi:10.1093/molbev/msae075)
Supplement: msae075_Supplementary_Data [file msae075_supplementary_data.zip › Supplementary information MBE with revisions.pdf]

Terrestrial birth and body size tune UCP1 functionality in seals

Michael J. Gaudry[a], Jane Khudyakov[b], Laura Pirard[c], Cathy Debier[c], Daniel Crocker[d], Paul G. Crichton[e], and Martin Jastroch[a]

[a]Department of Molecular Biosciences, The Wenner-Gren Institute, Stockholm University, Stockholm, Sweden.
[b]Department of Biological Sciences, University of the Pacific, Stockton, CA, USA.
[c]Louvain Institute of Biomolecular Science and Technology, Université catholique de Louvain, Louvain-la-Neuve, Belgium.
[d]Department of Biology, Sonoma State University, Rohnert Park, CA, USA.
[e]Biomedical Research Centre, Norwich Medical School, University of East Anglia, Norwich, UK.
* Martin Jastroch
Email: martin.jastroch@su.se

Supplementary information

Supplementary Figure 1

Supplementary data file 1 – RNA seq alignment

Supplementary data file 2 – UCP1 PAML alignment

Supplementary Table 1

Supplementary Table 2

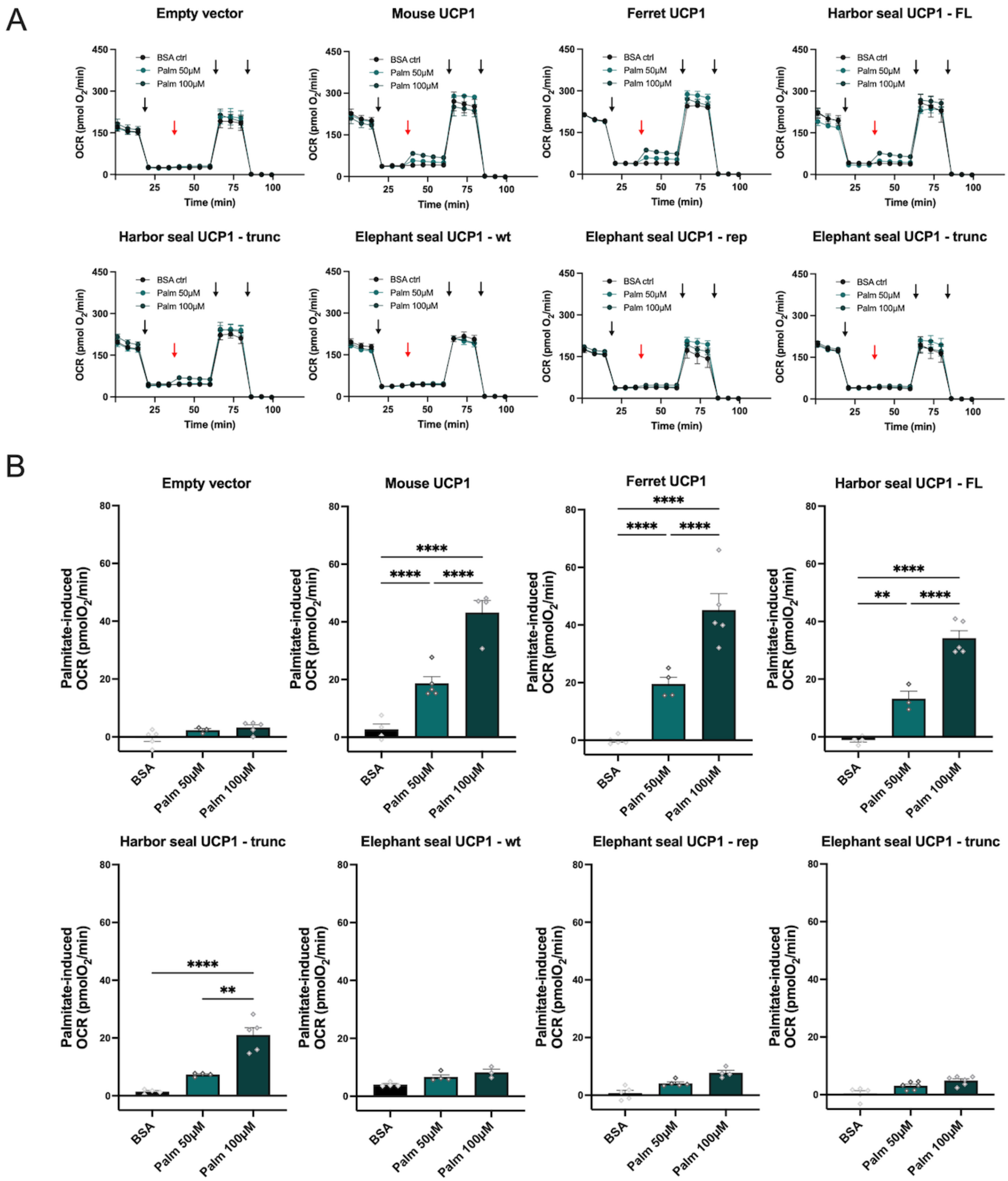

Supplementary Figure 1. Plate-based respirometry of transfected HEK293 cells confirms that elephant seal UCP1 is not thermogenic. A) Oxygen consumption rate (OCR) traces with (oligomycin, DNP, rotenone + antimycin A) injection points indicated with black arrows and UCP1 activator, palmitate (50 or 100µM), or BSA vehicle control injections indicated with red arrows. B) Palmitate-induced OCRs have been corrected for non-mitochondrial respiration. Data are mean ± s.e.m. N=3-6 from 3 independent runs. Statistical significances are denoted by **p<0.01, ****p<0.0001, one-way ANOVA with Tukey's post-hoc test.

Supplementary Table 1. Accession numbers containing UCP1 coding sequences used for selection pressure analyses. See supplementary data file 2 for alignment.

**Species name**	**Common name**	**Accession number**
Arctocephalus gazella	Antarctic fur seal	UIRR01000066.1
Arctocephalus townsendi	Guadalupe fur seal	JAQQSM010000002.1
Callorhinus ursinus	Northern fur seal	QLOG01010939.1
Eumetopias jubatus	Steller sea lion	SBAQ01004386.1
Halichoerus grypus	Grey seal	JAAXOB010005045.1
Leptonychotes weddellii	Weddell seal	APMU01141180.1; APMU01115166.1
Mirounga angustirostris	Northern elephant seal	PITE01002540.1
Mirounga leonina	Southern elephant seal	JAAMPH010000052.1
Mustela putorius furo	Ferret	AEYP01069989.1
Neomonachus schauinslandi	Hawaiian monk seal	NINY02000002.1
Odobenus rosmarus divergens	Walrus	ANOP01028105.1
Phoca vitulina	Harbor seal	RXNX01005441.1
Pusa hispida	Ringed seal	CAMRHF010000226.1
Zalophus californianus	California sea lion	PISZ01002091.1

Supplementary Table 2. Birth weight data and references for Figure 3B.

Species with *UCP1* pseudogenes

**Species**	**Birth weight (g)**	**Log birth weight**	**Reference**
Dasypus novemcinctus	80	1.903	Sacher and Staffeldt 1974
Bradypus variegatus	340	2.531	Hayssen et al. 1993
Choloepus hoffmanni	375	2.574	Hayssen et al. 1993
Cyclopes didactylus	364.3	2.561	Hayssen et al. 1993
Procavia capensis	200	2.301	Sacher and Staffeldt 1974
Elephas maximus	93000	4.968	Leitch et al. 1958
Loxodonta africana	120000	5.079	Sacher and Staffeldt 1974
Dugong dugon	27500	4.439	Marsh et al. 1984
Trichechus manatus latirostris	40000	4.602	Odell 2009
Balaena mysticetus	1000000	6.000	George et al. 2018
Balaenoptera acutorostrata	305000	5.484	McHuron et al. 2023
Balaenoptera bonaerensis	509800	5.707	Best and Lockyer 2002
Balaenoptera physalus	1500000	6.176	Leitch et al. 1958
Orcinus orca	153800	5.187	Clark et al. 2000
Tursiops truncatus	20000	4.301	Sacher and Staffeldt 1974
Delphinapterus leucas	88900	4.949	Robeck et al. 2005
Physeter macrocephalus	1016000	6.007	McHuron et al. 2023
Sus scrofa	1030	3.013	Sacher and Staffeldt 1974
Mirounga angustirostris	39850	4.600	Lee et al. 1991
Mirounga leonina	40600	4.609	Purvis and Harvey 1995
Equus asinus	22500	4.352	Gürcan et al. 2022
Equus caballus	53000	4.724	Sacher and Staffeldt 1974
Manis pentadactyla	130	2.114	Wu et al. 2020

Species with intact *UCP1* genes

Species	Birth weight (g)	Log birth weight	Reference
Echinops telfairi	8	0.903	Godfrey and Oliver 1978
Elephantulus edwardii	9	0.954	Stuart et al. 2003
Orycteropus afer	1525	3.183	Mutlow and Mutlow 2008
Bos grunniens	18000	4.255	Sacher and Staffeldt 1974
Bos taurus	25000	4.398	Sacher and Staffeldt 1974
Bubalus bubalis	34000	4.531	Leitch et al. 1958
Capra hircus	2180	3.338	Sacher and Staffeldt 1974
Ovis aries	3000	3.477	Sacher and Staffeldt 1974
Camelus ferus	35000	4.544	Chapman 1985
Vicugna pacos	7300	3.863	Grund et al. 2018
Choeropsis liberiensis	6750	3.829	Leitch et al. 1958
Canis lupus familiaris	404	2.606	Purvis and Harvey 1995
Acinonyx jubatus	530	2.724	Beekman et al. 1999
Felis catus	97.3	1.988	Purvis and Harvey 1995
Panthera tigris altaica	1300	3.114	Sacher and Staffeldt 1974
Panthera uncia	450	2.653	Hemmer 1972
Mustela putorius furo	9.53	0.979	Purvis and Harvey 1995
Neovison vison	11.1	1.045	Matthiesen et al. 2010
Odobenus rosmarus	63550	4.803	Lee et al. 1991
Zalophus californianus	8450	3.927	McHuron et al. 2023
Eumetopias jubatus	20550	4.313	McHuron et al. 2023
Arctocephalus townsendi	5500	3.740	McHuron et al. 2023
Arctocephalus gazella	5300	3.724	McHuron et al. 2023
Leptonychotes weddellii	24000	4.380	Lee et al. 1991
Phoca vitulina	9500	3.978	Lee et al. 1991
Halichoerus grypus	15450	4.189	McHuron et al. 2023
Pusa hispida	4850	3.686	McHuron et al. 2023
Monachus schauinslandi	15640	4.194	Wirtz 1986
Ailuropoda melanoleuca	363.7	2.561	Zhu et al. 2001
Ursus maritimus	590	2.771	Leitch et al. 1958
Megaderma lyra	7.5	0.875	Hayssen et al. 1993
Eidolon helvum	50	1.699	Leitch et al. 1958
Pteropus vampyrus	133.3	2.125	Kunz and Hood 2000

Rhinolophus ferrumequinum	6.2	0.792	Purvis and Harvey 1995
Eptesicus fuscus	3.54	0.549	Purvis and Harvey 1995
Myotis lucifugus	2.08	0.318	Leitch et al. 1958
Erinaceus europaeus	16.1	1.207	Purvis and Harvey 1995
Sorex araneus	0.5	-0.301	Hayssen et al. 1993
Suncus murinus	2.1	0.322	Gaillard et al. 1997
Diceros bicornis	34000	4.531	Lee et al. 1991
Ceratotherium simum	55000	4.740	Lee et al. 1991
Tapirus indicus	7000	3.845	Sacher and Staffeldt 1974
Ochotona princeps	10.2	1.009	Purvis and Harvey 1995
Oryctolagus cuniculus	37.6	1.575	Purvis and Harvey 1995
Aotus nancymaae	94	1.973	Smith et al. 2017
Callithrix jacchus	30.2	1.480	Smith and Leigh 1997
Saimiri boliviensis	113.61	2.055	Mulholland et al. 2019
Cercocebus atys	590	2.771	Smith and Leigh 1997
Macaca fascicularis	339.6	2.531	Smith and Leigh 1997
Macaca mulatta	540	2.732	Sacher and Staffeldt 1974
Macaca nemestrina	473	2.675	Lee et al. 1991
Mandrillus leucophaeus	722	2.859	Smith and Leigh 1997
Nasalis larvatus	600	2.778	Smith and Leigh 1997
Papio anubis	950	2.978	Lee et al. 1991
Papio hamadryas	443	2.646	Sacher and Staffeldt 1974
Daubentonia madagascariensis	115	2.061	Glander et al. 1994
Otolemur garnettii	52	1.716	Smith and Leigh 1997
Gorilla gorilla gorilla	1750	3.243	Sacher and Staffeldt 1974
Homo sapiens	3660	3.563	Sacher and Staffeldt 1974
Pan paniscus	1400	3.146	Lee et al. 1991
Pan troglodytes	1740	3.241	Purvis and Harvey 1995
Eulemur flavifrons	67.9	1.832	Quintard et al. 2017
Eulemur macaco	56.85	1.755	Smith and Leigh 1997
Tarsius syrichta	25	1.398	Smith and Leigh 1997
Microcebus murinus	5.9	0.771	Smith and Leigh 1997
Cavia porcellus	97	1.987	Sacher and Staffeldt 1974
Chinchilla lanigera	49.5	1.695	Dzierzanowska-Goryn et al. 2023
Mesocricetus auratus	1.8	0.255	Sacher and Staffeldt 1974
Microtus agrestis	2.9	0.462	Gaillard et al. 1997
Peromyscus maniculatus	1.6	0.204	Purvis and Harvey 1995
Jaculus jaculus	2	0.301	Millar et al. 1977

Heterocephalus glaber	1.5	0.176	Jarvis 1991
Mus musculus	1.5	0.176	Sacher and Staffeldt 1974
Rattus norvegicus	4.92	0.692	Sacher and Staffeldt 1974
Octodon degus	14.6	1.164	Reynolds and Wright 1979
Spermophilus tridecemlineatus	2.6	0.415	Leitch et al. 1958
Tupaia belangeri chinensis	10	1.000	Martin 1968

References for birth weights

Beekman SPA, Kemp B, Louwman HCM, Colenbrander B. 1999. Analyses of factors influencing the birth weight and neonatal growth rate of Cheetah (*Acinonyx jubatus*) cubs. *Zoo Biology.* 18:129–139.

Best PB, Lockyer CH. 2002. Reproduction, growth and migrations of sei whales *Balaenoptera borealis* off the west coast of South Africa. *Afr J Mar Sci.* 24:111–133.

Chapman MJ. 1985. Bactrian Camels–World animal review. p 14-19.

Clark ST, Odell DK, Lacinak CT. 2000. Aspects of Growth in Captive Killer Whales (*Orcinus orca*). *Mar Mamm Sci.* 16:110–123.

Dzierzanowska-Goryn D, Brzozowski M, Goral-Radziszewska K. 2014. Young chinchillas weight gain, depending on their body mass at birth. *Ann Wars Univ Life Sci – SGGW, Anim Sci.* 53.

Gaillard J-M, Pontier D, Allaine D, Loison A, Herve J-C, Heizman A. 1997. Variation in growth form and precocity at birth in eutherian mammals. *Proc R Soc B: Biol Sci.* 264:859–868.

George JC, Rugh D, Suydam R. 2018. Bowhead Whale: Balaena mysticetus. In: Würsig B, Thewissen JGM, Kovacs KM, editors. Encyclopedia of Marine Mammals (Third Edition). Academic Press. p. 133–135.

Godfrey GK, Oliver WLR. 1978. The reproduction and development of the pigmy hedgehog tenrec, *Echinops telfairi*. *Dodo J Jersey Wildl Preserv Trust* 15:38–51.

Grund S, Vogel M, Mülling CKW. 2018. Morphometric evaluation of the growth of Alpacas (*Vicugna pacos*) from birth to 36 months of age. *Small Rumin Res.* 166:61–65.

Gürcan EK, Genç S, Kaplan S, ÖzdiL F, Ünal EÖ, Bulut H, Yatkin S, Arat S, Soysal Mİh. 2022. Determination of the morphometric characteristics of donkey (*Equus asinus*) populations reared in Turkey. *Turkish J Vet Anim Sci.* 46:445–456.

Hayssen VD, Tienhoven Ari Van, Tienhoven Ans Van. 1993. Asdell's Patterns of Mammalian Reproduction: A Compendium of Species-specific Data. Cornell University Press

Kunz TH, Hood WR. 2000. 10 - Parental Care and Postnatal Growth in the Chiroptera. In: Crichton EG, Krutzsch PH, editors. Reproductive Biology of Bats. London: Academic Press. p. 415–468.

Lee PC, Majluf P, Gordon IJ. 1991. Growth, weaning and maternal investment from a comparative perspective. *J Zool.* 225:99–114.

Leitch I, Hytten FE, Billewicz WZ. 1959. The maternal and neonatal weights of some Mammalia. *Proc Zool Soc Lond* 133:11–28.

Marsh H, Heinsohn GE, Marsh LM. 1984. Breeding Cycle, Life History and Population Dynamics of the Dugong, *Dugon dugon* (Sirenia: Dugongidae). *Aust J Zool.* 32:767–788.

Martin RD. 1968. Reproduction and Ontogeny in tree-shrews (Tupaia belangeri), with reference to their general behaviour and taxonomic relationships. *Z Tierpsychol.* 25:505–532.

Matthiesen CF, Blache D, Thomsen PD, Tauson A-H. 2010. Feeding mink (*Neovison vison*) a protein-restricted diet during pregnancy induces higher birth weight and altered hepatic gene expression in the F2 offspring. *Br J Nutr.* 104:544–553.

McHuron EA, Adamczak S, Costa DP, Booth C. 2023. Estimating reproductive costs in marine mammal bioenergetic models: a review of current knowledge and data availability. *Conserv Physiol.* 11:coac080.

Millar JS. 1977. Adaptive Features of Mammalian Reproduction. *Evolution.* 31:370–386.

Mulholland MM, Williams LE, Abee CR. 2020. Neonatal activity and state control differences among three squirrel monkey subspecies (*Saimiri sciureus sciureus*, *S. boliviensis boliviensis*, and *S. boliviensis peruviensis*). *Am J Primatol.* 82:e23048.

Mutlow AG, Mutlow H. 2008. Caesarian Section and Neonatal Care in the Aardvark (*Orycteropus afer*). *J Zoo Wildlife Med.* 39:260–262.

Odell DK. 2009. Sirenian Life History. In: Perrin WF, Würsig B, Thewissen JGM, editors. Encyclopedia of Marine Mammals (Second Edition). London: Academic Press. p. 1019–1021.

Purvis A, Harvey PH. 1995. Mammal life-history evolution: a comparative test of Charnov's model. *J Zool.* 237:259–283.

Quintard B, Petit T, Lefaux B. 2017. Hand-rearing the critically endangered blue-eyed black lemur (*Eulemur flavifrons*): milk formula, feeding and socialisation protocols. *J Zoo Aquar Res.* 5:76–81.

Reynolds TJ, Wright JW. 1979. Early postnatal physical and behavioural development of degus (*Octodon degus*). *Lab Anim.* 13:93–100.

Robeck TR, Monfort SL, Calle PP, Dunn JL, Jensen E, Boehm JR, Young S, Clark ST. 2005. Reproduction, Growth and Development in Captive Beluga (*Delphinapterus leucas*). *Zoo Biol.* 24:29–49.

Sacher GA, Staffeldt EF. 1974. Relation of Gestation Time to Brain Weight for Placental Mammals: Implications for the Theory of Vertebrate Growth. *Am Nat.* 108:593–615.

Sherman PW, Jarvis JUM, Alexander RD. 2017. The Biology of the Naked Mole-Rat. Princeton University Press

Smith RJ, Leigh SR. 1998. Sexual dimorphism in primate neonatal body mass. *J Hum Evol.* 34:173–201.

Smith TD, Muchlinski MN, Bucher WR, Vinyard CJ, Bonar CJ, Evans S, Williams LE, DeLeon VB. 2017. Relative tooth size at birth in primates: Life history correlates. *Am J Phys Anthropol.* 164:623–634.

Wirtz WO II. 1968. Reproduction, Growth and Development, and Juvenile Mortality in the Hawaiian Monk Seal. *J Mammal.* 49:229–238.

Wu S, Sun NC-M, Zhang F, Yu Y, Ades G, Suwal TL, Jiang Z. 2020. Chapter 4 - Chinese pangolin Manis pentadactyla (Linnaeus, 1758). In: Challender DWS, Nash HC, Waterman C, editors. Pangolins. Biodiversity of World: Conservation from Genes to Landscapes. Academic Press. p. 49–70.

Zhu X, Lindburg DG, Pan W, Forney KA, Wang D. 2001. The reproductive strategy of giant pandas (*Ailuropoda melanoleuca*): infant growth and development and mother–infant relationships. *J Zool.* 253:141–155.
